# Moral asymmetries in judgments of agency withstand ludicrous causal deviance

**DOI:** 10.3389/fpsyg.2015.01380

**Published:** 2015-09-15

**Authors:** Paulo Sousa, Colin Holbrook, Lauren Swiney

**Affiliations:** ^1^Institute of Cognition and Culture, Queen’s University Belfast, Belfast, UK; ^2^Department of Anthropology, Center for Behavior, Evolution, and Culture, University of California, Los Angeles, Los Angeles, CA, USA; ^3^Institute of Cognitive and Evolutionary Anthropology, School of Anthropology, University of Oxford, Oxford, UK

**Keywords:** blame, credit, action, intentional action, causal deviance, moral judgments

## Abstract

Americans have been shown to attribute greater intentionality to immoral than to amoral actions in cases of causal deviance, that is, cases where a goal is satisfied in a way that deviates from initially planned means (e.g., a gunman wants to hit a target and his hand slips, but the bullet ricochets off a rock into the target). However, past research has yet to assess whether this asymmetry persists in cases of extreme causal deviance. Here, we manipulated the level of mild to extreme causal deviance of an immoral versus amoral act. The asymmetry in attributions of intentionality was observed at all but the most extreme level of causal deviance, and, as we hypothesized, was mediated by attributions of blame/credit and judgments of action performance. These findings are discussed as they support a multiple-concepts interpretation of the asymmetry, wherein blame renders a naïve concept of intentional action (the outcome matches the intention) more salient than a composite concept (the outcome matches the intention *and* was brought about by planned means), and in terms of their implications for cross-cultural research on judgments of agency.

## Introduction

Reasoning about causes is a fundamental aspect of human cognition. It is unlikely that causal cognition is a homogeneous phenomenon; the human mind is likely to have different causal competencies, which draw on different causal concepts or deploy similar causal concepts differently depending on context (see, e.g., Sperber et al., 1995; Danks et al., 2014). Here, we focus on the causal competencies deployed in the understanding of agency, both in terms of judging that an event is an intentional action, and judging that an event is an action at all. Reasoning about causes is fundamental to such judgments because they require an understanding of the causal links between mental states, bodily movements and succeeding events in the world, as well as an understanding of various interfering factors. In this article, we investigate how causal cognition drives judgments of agency, and how this varies depending on moral context.

We investigate judgments of agency in relation to events involving causal deviance, that is, events involving causal chains that are initiated by an agent, that lead to the satisfaction of the agent’s intention, but that do not follow the agent’s plan. For example, an agent intends to hit a target with his rifle, and indeed does hit the target, but not in the planned manner—instead of the bullet going directly into the target, it ricochets off a rock and into the target.

Our investigation relates to current work on asymmetries in judgments of intentionality indicating that people (primarily westerners) tend to judge the same type of action to be intentional in moral contexts but unintentional in other contexts (for a review, see Cova, 2015). In our previous work with an American sample, we demonstrated this asymmetry in cases of mild causal deviance, and we argued for a multiple-concepts explanation in which distinct concepts of intentional action are selected in different contexts depending on considerations of blame and credit (see Sousa and Holbrook, 2010; for similar approaches, see Mele and Cushman, 2007; Nichols and Ulatowski, 2007; Cushman and Mele, 2008; Cova et al., 2012; Lanteri, 2013). Here, we extend our investigation to cases of more extreme causal deviance in order to explore further issues concerning judgments of action performance (i.e., judgments of whether an event is the action of an agent). With this extension, we shall explore the potential boundary conditions of the asymmetry in judgments of intentionality by experimentally probing the extent to which the asymmetry persists in conditions of extreme causal deviance.

In the first section, we characterize our multiple-concepts approach to the asymmetry in judgments of intentionality in cases of mild causal deviance and its relation to attributions of blame and credit. In the second section, drawing from our approach, we characterize our main hypotheses regarding cases of greater causal deviance. In the third section, we report an exploratory study of the extent to which judgments of intentionality and action performance occur at different levels of mild to extreme causal deviance and across amoral versus immoral contexts, and discuss its results in terms of our multiple-concepts approach and alternative explanatory models. We then point out some of the limitations of our current results and delineate some future avenues of research, before concluding with a general remark on pursuing cross-cultural research on the topic of causal cognition and judgments of agency.

### Polysemy Masks Competing Concepts of Intentional Action

To illustrate the asymmetry in judgments of intentionality in cases of mild causal deviance, consider the following parallel scenarios involving amoral versus immoral shooting:

John desperately wants to win the rifle contest [wants to have more money]. He knows that he will only win the contest if he hits the bull’s-eye [that he will inherit a lot of money when his aunt Mary dies]. To win the contest [to kill his aunt], John raises his brand-new rifle and aims at the bull’s-eye [Mary’s heart], which is 150 feet away. John has never fired a gun before, and he has no natural talent for this type of thing. His hand slips on the barrel of the gun, and the shot goes wild… The bullet hits a rock situated 80 feet in front of John. He assumes he has completely missed the target. But what a surprise: the bullet actually bounces off the rock and goes directly into the bull’s-eye [Mary’s heart].

When asked whether the amoral action *hitting the bull’s eye* is intentional, the majority of participants say that it is not intentional; when asked whether the immoral action *hitting the aunt’s heart* is intentional, the great majority of participants affirm that it is intentional^1^.

Our multiple-concepts approach to the asymmetry in judgments of intentionality claims that the expression “intentional action” (or “acting intentionally”) is polysemous: there are stable associations in people’s minds between such phrases and distinct concepts of intentional action, although there may be substantial individual variation in terms of the relative strength of each association^2^. In particular, we claim that two distinct concepts of intentional action play a role in people’s answers to the intentionality question in the above scenarios. In the first concept, which we call the *composite* concept, an action A of an agent S is considered intentional *only if* S had the intention to A and the causal chain constituting A follows S’s plan to A, using “plan” in the specific sense of S’s representation of the intended steps to satisfy S’s intention to A (for a more complete characterization of this concept, see Sousa and Holbrook, 2010). With this concept in mind, in both amoral and immoral contexts, the action *hitting the target* is to be considered *not* intentional, since it does not follow S’s plan to hit the target with the bullet going directly into the target (i.e., without the ricochet). In the second concept, which we call the *naïve* concept, an action A of an agent S is considered intentional *if and only if* S had the intention to A. With this concept in mind, in both amoral and immoral contexts, the action *hitting the target* is to be considered intentional, since it satisfies S’s intention to hit the target.

Consistent with the psychological reality of this polysemy, when participants judge the action in question to be unintentional, they justify their judgment by emphasizing that plan-following is a necessary condition, in accord with our postulated composite concept. For example (see Sousa and Holbrook, 2010), they say:

“…the means by which he hit the bull’s-eye wasn’t planned, and so the unintentional means of hitting the bull’s-eye qualifies the hit as unintentional.”

“It is unintentional that the bullet bounced from the rock to the heart.”

Moreover, in accord with our postulated naïve concept, when participants judge the action in question to be intentional, they justify their answer by emphasizing that plan-following is not a necessary condition and suggesting that intention satisfaction is a sufficient one. For example (see Sousa and Holbrook, 2010), they say:

“John’s goal was to hit the bull’s-eye. He did not hit it in the manner in which he intended, but his intention to hit the bull’s-eye never changed.”

“… because the effect of hitting his aunt’s heart was still there. He accomplished his goal, even if it was indirectly.”

Crucially, the relative salience of whether the intention was satisfied (the naïve concept) as opposed to whether the intention was satisfied by planned means (the composite concept) tracks the relative importance of these factors to concepts of blame/credit across immoral and amoral contexts. For the attribution of blame in an immoral context such as the killing of the aunt, the immoral intentions (i.e., the intentions closely connected with immoral motivations), rather than the manner in which they are satisfied, are the most relevant factor, since the type of decision that the agent makes (e.g., to commit murder) determines the moral evaluation of the agent. Thus, for the great majority, the naïve concept is more salient in the immoral context, leading them to respond to the intentionality question in terms of whether the intention was satisfied—and accordingly to judge the action to be intentional. By contrast, for the attribution of credit in an amoral context such as the rifle contest, people respond as if they are divided between the two competing naïve and composite concepts (for more details, see Sousa and Holbrook, 2010). Many take the manner in which the intention is satisfied as fundamental, since for them whether the goal is achieved in a planned and skillful (rather than merely lucky) manner determines the merit of the agent. For these individuals, the composite concept is the most relevant. However, many people also discount the manner in which the amoral intention is satisfied, and therefore favor the naïve concept. Thus, overall, fewer people in the amoral context will interpret the intentionality question in terms of the naïve concept and judge the action to be intentional—hence, the asymmetry in judgments of intentionality.

### Extreme Causal Deviance

With the composite concept of intentional action in mind, any deviance from the planned means of achieving the goal should lead one to judge an agent’s action to be unintentional. In cases where the naïve concept is in mind, however, it is less clear what effect different levels of causal deviance might exert on judgments of intentionality. For example, returning to John’s killing of his aunt Mary as an example, suppose that John’s bullet misses Mary by a mile, but the report of the rifle stampedes a herd of wild pigs that tramples Mary to death (cf. Davidson, 1980). Insofar as such extreme deviance precludes the categorization of the events leading to Mary’s death as an action of John, let alone as an intentional action of John, most people may deny that John killed Mary intentionally—even with the naive concept in mind. In other words, the denial of intentionality in this case would be due to a problem with the superordinate folk concept of action; presumably, if not *S’s A*, then not *S’s intentional A*. Thus, it is possible that in extreme cases of causal deviance the asymmetry in judgments of intentionality would vanish, since, in both immoral and amoral contexts, most people would deny intentionality.

A few remarks about the complementary folk concepts of action and agent (i.e., the doer of the action) are in order to explicate the above hypothesis. According to the folk concept of action, an action A is an event (i) whose description fits the scheme “what agent S did was …” and (ii) whose agent S is interpreted as the causal producer of the causal chain constituting the action A^3^. Consider whether the following sentences encode action concepts in this sense:

(a) The door is open (– event; – action)
(b) The door closed (+ event; – action)
(c) The wind closed the door (+ event; + action; – animate agent)
(d) John opened the door (+ event; + action; + animate agent)

Sentence (a) describes a state, which by definition cannot be an event; therefore it does not encode an action concept. Sentence (b) describes something that happened to the door (i.e., the closing of the door was not something that the door did), and therefore does not encode an action concept either. Sentences (c) and (d) describe what the wind and John did (i.e., close or open the door) and these entities can be interpreted as causal producers of the door closing or opening; therefore, these sentences encode two different action concepts.

Sentences encoding action concepts are often neutral with regards to the intentionality of the action^4^, although they preclude intentionality features when they involve an inanimate agent, and they encode intentionality when its verb encodes intentionality. Consider the following sentences:

(e) John killed Mary (+ animate agent; ± intentionality)
(f) The wind closed the door (– animate agent)
(g) John murdered Mary (+ animate agent; + intentionality)

Sentence (e) is neutral with regards to the intentionality of the action—a sentence like “John killed Mary intentionally (or unintentionally)” would be intelligible. Sentence (f) precludes intentionality features because in involves an inanimate agent—strictly speaking, a sentence like “the wind closed the door intentionally (or unintentionally)” is not intelligible. Finally, sentence (g) encodes intentionality because the verb “to murder” encodes intentionality—strictly speaking, a sentence like “John murdered Mary unintentionally” is not intelligible, and a sentence like “John murdered Mary intentionally” is redundant.

Ordinary action descriptions in the sense of action we characterized may incorporate an unfolding causal chain of events^5^. Consider the following sequence of events: John opens the door; Mary, who is inside the room, startles; Mary suffers a heart attack; Mary dies. One can describe this sequence of events by saying simply, “John killed Mary.” This description refers to only one action (John’s killing of Mary), an action incorporating all the events of the sequence (John’s opening the door, Mary’s startle, her heart attack, and her death)^6^. Also, consider the following sequence of events: John pulls the trigger; the gun discharges; the bullet goes directly into Mary’s heart; Mary dies. One can describe this sequence of events by saying simply, “John killed Mary.” This description refers to only one action (John’s killing Mary), an action incorporating all the events of the sequence (John’s pulling the trigger, the gun’s discharge, the bullet going into Mary’s heart, and Mary’s death). Finally, consider the exact same sequence of events as the last one, except that the bullet ricochets off the rock and directly into Mary’s heart. Again, one can describe this sequence of events by saying simply, “John killed Mary.” This description refers to only one action (John’s killing Mary), an action incorporating all the events of the sequence (John’s pulling the trigger, the gun’s discharge, the bullet bouncing off the rock and going into Mary’s heart, and Mary’s death).

The folk concept of action may imply constraints on the amount and types of constitutive events an agent’s action can incorporate. Plausibly, the longer the unfolding causal chain, the less one may envisage the original agent as the causal producer of the final effect of the chain, and hence the less one may think that the final effect could be part of an action of the original agent. For instance, in relation to the startle example above, suppose an extended sequence of events were to transpire: Mary suffers a heart attack; still alive, Mary is sent to the hospital; the ambulance suffers a flat tire and crashes due to a nail in the road, further injuring Mary; Mary dies due to the crash injuries. Can Mary’s death still be described as part of an action “John killed Mary,” or is it rather more appropriate to say that the crash injuries killed Mary? Further, it may be the case that events in the unfolding causal chain that involve animate agents may make one think less of the original animate agent as the causal producer of the final effect of the chain. For instance, returning to the shooting examples above, suppose that the bullet misses Mary by a mile; the shot stampedes a herd of wild pigs; the wild pigs trample Mary; Mary dies. Would Mary’s death still be described as part of “John killed Mary,” or would people be more inclined to say that the pigs killed Mary?

Returning to our hypothesis and the issue of causal deviance, the startle scenarios described above do not qualify as instances of causal deviance, since John did not intend or have a plan to bring about Mary’s death. By contrast, the ricochet death scenario constitutes a case of mild causal deviance, and the stampede death scenario constitutes a case of extreme causal deviance. We propose that the naïve concept of intentionality should not be invoked in cases of extreme causal deviance to the extent that constraints on the amount and/or types of events that an action of an agent could incorporate deter participants from viewing the event as an action of the agent^7^. Accordingly, in cases of extreme causal deviance, the asymmetry in judgments of intentionality may disappear along with the perception that the agent performed the action.

On the other hand, it is possible that the effect of causal deviance on judgments of action performance might differ by moral context, with more people judging the event as an action of the agent in immoral contexts than in amoral ones. It is plausible that, in the immoral context, the attribution of blame renders the agent more salient as the causal producer of the intended outcome, making deviant causal chains more tolerable and judgments of action performance more resilient. If this were the case, we might expect the asymmetry in ratings of intentionality to persist even at high levels of causal deviance, in parallel with an asymmetry in judgments of action performance.

Whether or not extreme causal deviance negates the asymmetry in intentionality judgments, our key point is that these judgments should closely track judgments of action performance. Similarly, to the extent that blame attributions inherently attract participants to the naïve concept of intentionality by highlighting the salience of the actor’s immoral intentions, we claim that the asymmetry in intentionality judgments should persist despite extreme causal deviance only to the extent that high attributions of blame persist.

## Study

To explore the aforementioned hypotheses, we presented people with scenarios describing five levels of causal deviance, from mild to quite extreme, and asked them to make judgments of blame/credit, action performance, and intentionality.

## Method

### Participants

Three hundred and ten participants were recruited via Craigslist.org to volunteer for an unpaid online study advertised as a “10-minute Action Survey” from regions across the United States. Four participants were removed prior to analysis for having provided incomplete responses, leaving a sample of 306 participants (48% female). Participation took place online.

### Design, Materials, and Procedures

All participants gave their informed consent to participate in the study. The protocol of the study was approved by the ethics committee of the School of History and Anthropology, Queen’s University, Belfast. The study was carried out following the guidelines and recommendations of the same committee. In a 2 (immoral versus amoral context) × 5 (deviance level) between-subjects design, each participant was presented with one of 10 vignettes (∼30 participants per vignette). The five immoral and amoral vignettes started with the following scenarios, respectively.

#### Immoral

For no particular reason, Sam wants to upset his neighbor. In order to so, he plans to break the neighbor’s beloved vase inherited from his grandmother. The vase is positioned in the neighbor’s front yard, 100 m away from Sam. He raises his rifle and aims at the center of the vase. Sam is completely sure about his decision, but he is not skilled with rifles. He pulls the trigger, but the shot goes wild.

#### Amoral

Fred wants to win a game. In order to do so, a vase has to be broken. The vase is positioned in a field, 100 m away from Fred. He raises his rifle and aims at the center of the vase. Fred wants to win the game, but he is not skilled with rifles. He pulls the trigger, but the shot goes wild.

All vignettes ended with, “The vase is broken.” *The neighbor is devastated* (or *Fred wins the game*). In between, one of the following five levels of causal deviance was described.

#### Level 1

However, the bullet bounces off a rock and hits the vase.

#### Level 2

However, the bullet bounces off a rock and hits the tire of a passing car. The car veers out of control into the *front yard* (or *field*) and strikes a post. The post falls onto a tree, breaking off a branch. The branch falls onto the vase.

#### Level 3

However, the bullet bounces off a rock and hits the tire of a passing car. The car veers out of control into the *front yard* (or *field*) and strikes a post. The post falls onto a tree, breaking off a branch. The branch falls onto an unsteady log, setting it in motion. The rolling log hits an old, forgotten mousetrap. The spring-loaded trap is launched into the air, landing right next to a squirrel. The squirrel is startled and runs. The squirrel accidentally bumps into the vase. The vase falls over and rolls several feet. The vase hits a pointy rock.

#### Level 4

However, the bullet bounces off a rock and hits the tire of a passing car. The car veers out of control into the *front yard* (or *field*) and strikes a post. The post falls onto a tree, breaking off a branch. The branch falls onto an unsteady log, setting it in motion. The rolling log hits an old, forgotten mousetrap. The spring-loaded trap is launched into the air, landing right next to a squirrel. The squirrel is startled and runs. The squirrel runs past a dog and the dog begins to chase the squirrel. The dog chases the squirrel around the *yard* (or *field*) for several minutes. While running, the dog slips and accidentally bumps into the vase. The vase falls over and rolls several feet. The vase hits a pointy rock.

#### Level 5

However, the bullet bounces off a rock and hits the tire of a passing car. The car veers out of control into the *front yard* (or *field*) and strikes a post. The post falls onto a tree, breaking off a branch. The branch falls onto an unsteady log, setting it in motion. The rolling log hits an old, forgotten mousetrap. The spring-loaded trap is launched into the air, landing right next to a squirrel. The squirrel is startled and runs. The squirrel runs toward a young boy and his father who are walking around the *neighborhood* (or *field*). The young boy begins to chase the squirrel back into the *yard* (or *field*). The squirrel soon runs out of sight. In frustration, the boy mindlessly picks up the vase and throws it in the direction of the squirrel.

For each scenario, three questions were asked in fixed order: (i) How much credit [blame] does Fred [Sam] deserve? (ii) Does it sound right to say that “Fred [Sam] broke the vase”? (iii) Does it sound right to say that “Fred [Sam] broke the vase intentionally”? As is usually done in the literature (see Knobe, 2003), the credit/blame question appeared first to offer participants a way of explicitly communicating their (moral) evaluation separately and to free them to pursue a literal answer to the subsequent questions. Otherwise, given the prototypical association between intentionality and blame, many participants may avoid saying that the immoral act is unintentional just because, if they were to say that, they would give the idea that they do not blame the immoral agent. Because we wanted to allow participants to clearly envisage the logical relation between the action performance and the intentionality questions, we positioned the former question before the latter.

All questions were answered on a 7-point Likert scale anchored as “0 = *None*; 3 = *Medium*; 6 = *Full*” for the credit/blame question, and as “0 = *Totally wrong*; 3 = *In between*; 6 = *Totally right*” for the action performance and intentional action questions. Participants were asked to justify their answer to each of the three questions, which they did by writing down their justifications in open response boxes.

## Results

### Judgments

Mean ratings of blame/credit, action performance, and intentionality by moral context and deviance level can be found in Table 1. Preliminary analyses showed that all three ratings of credit/blame, action, and intentionality were positively intercorrelated, *r*s ranging from 0.55 to 0.65, *p*s < 0.001.

**TABLE 1:** **Mean Ratings of Credit/Blame, Action, and Intentionality by Moral Context and Deviance Condition**.

****	**Level 1 *Mean (SD)***	**Level 2 *Mean (SD)***	**Level 3 *Mean (SD)***	**Level 4 *Mean (SD)***	**Level 5 *Mean (SD)***
*Immoral Context*					
Blame	5.87 (0.43)^a^	5.89 (0.32)^a^	5.23 (1.25)^a,b^	5.10 (1.60)^b,c^	4.73 (1.95)^b,c^
Action	5.77 (0.67)^a^	4.43 (1.87)^b^	4.30 (1.84)^b,c^	3.52 (1.88)^c^	2.53 (2.16)^d^
Intentionality	5.71 (1.19)^a^	5.14 (1.33)^a,b^	4.67 (2.06)^b^	4.26 (2.37)^b^	2.80 (2.37)^c^
*Amoral context*					
Credit	3.93 (2.13)^a^	3.06 (2.50)^a,b^	2.59 (2.47)^b^	2.08 (2.49)^b^	2.55 (2.34)^b^
Action	4.87 (1.57)^a^	2.81 (2.35)^b^	2.90 (2.21)^b^	1.53 (1.89)^c^	1.72 (2.00)^c^
Intentionality	4.30 (1.97)^a^	3.00 (2.40)^b^	2.90 (2.61)^b,c^	1.86 (2.27)^c,d^	1.93 (2.33)^b,c^

N = 306. Row means that do not share a superscript letter are significantly different with alpha at 0.05.

**FIGURE 1:**
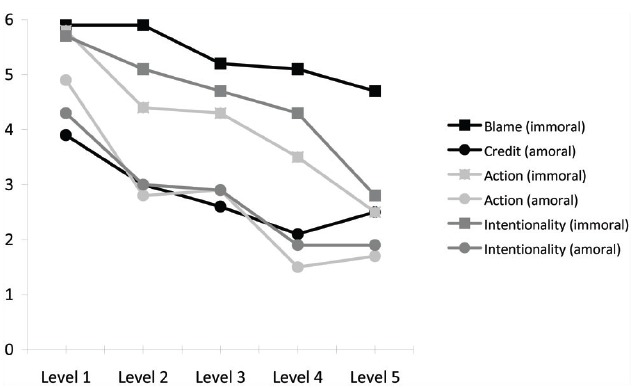
**Attributions of credit/blame, action and intentionality under increasing levels of causal deviance**.

A multivariate two-way between-subjects ANOVA revealed a significant main effect of moral context on all three ratings, *F*(3,294) = 44.52, *p* < 0.001, ${\text{η}}_{p}^{2}$ = 0.31. In line with previous findings, participants in the immoral condition provided higher ratings of both blame/credit and intentionality for the action of breaking the vase; ratings of action performance were also higher in the immoral condition (see Table 2)^8^. The model also revealed a significant main effect of deviance condition, with ratings of all three measures diminishing with successive increases in the level of causal deviance, *F*(4,296) = 27.47, *p* < 0.001, ${\text{η}}_{p}^{2}$ = 0.27 (see Figure 1). However, the drop was least sizable in the blame measure. There was no significant Context × Deviance interaction, *p* = 0.186.

**TABLE 2:** **Mean Ratings of Credit/Blame, Action, and Intentionality by Moral Context**.

****	**Immoral *Mean (SD)***	**Amoral *Mean (SD)***	***F***	***p***	**${\text{η}}_{p}^{2}$**	**95% CI**
Blame/credit	5.36 (1.35)	2.82 (2.45)	124.98	<0.001	0.29	–2.99, –2.09
Action	4.11 (2.05)	2.72 (2.32)	30.76	<0.001	0.09	–1.88, –0.90
Intentionality	4.51 (2.51)	2.77 (2.46)	43.51	<0.001	0.13	–2.26, –1.22

N = 306. These means pool across the deviance conditions of the entire sample.

Follow-up analyses confirmed that, in the first four causal deviance conditions, all three ratings significantly differed between the amoral versus immoral conditions, with *p*s ranging from 0.01 to –0.000001, ${\text{η}}_{p}^{2}$ values from 0.11 to –0.38, and confidence intervals never crossing 0. However, there was no significant difference between the amoral and immoral conditions at the fifth and most extreme level of causal deviation with respect to ratings of action performance, *p* = 0.14, ${\text{η}}_{p}^{2}$ = 0.04, 95% CI = (–1.90, 0.28), or intentionality, *p* = 0.16, ${\text{η}}_{p}^{2}$ = 0.03, 95% CI = (–2.09, 0.36). In the case of credit/blame judgments, the effect of moral condition remained robust at the most extreme level of causal deviance, *p* < 0.001, ${\text{η}}_{p}^{2}$ = 0.21, 95% CI = (–3.30, –1.06).

Next, we conducted a series of mediation tests to assess the contributions of attributions of credit/blame and of action performance to the heightened ratings of intentionality. We utilized the bias-corrected bootstrapping procedure (5,000 samples) found in the INDIRECT macro for SPSS (Preacher Hayes, 2008).

We first assessed the influence of attributions of credit/blame on intentionality ratings by entering moral context as the independent variable, ratings of credit/blame as the potential mediator, and intentionality ratings as the outcome variable, controlling for action performance attributions as a covariate. In the model, attributions of credit/blame fully mediated the effects of the morality manipulation on ratings of intentionality. The direct effect of moral context on intentionality (*b* = 0.85, SE = 0.22, *p* < 0.001) was no longer significant with ratings of credit/blame included in the model (*b* = 0.24, SE = 0.24, *p* = 0.31), whereas the indirect effect of credit/blame on rated intentionality was significant (*b* = 0.31, SE = 0.06, *p* < 0.001), and the confidence intervals did not overlap with 0 [95% CI = (0.34, 0.93)].

Then, we assessed the contribution of attributions of action performance to intentionality ratings by entering action performance attribution as the potential mediator. In this model, the direct effect of moral context on intentionality (*b* = 1.74, SE = 0.26, *p* < 0.001) was approximately halved, yet remained highly significant (*b* = 0.85, SE = 0.22, *p* < 0.001). The indirect effect of action attribution on rated intentionality was significant (*b* = 0.65, SE = 0.05, *p* < 0.001), and the confidence intervals did not overlap with 0 [95% CI = (0.58, 1.25)]. Thus, judgments of action performance partially mediated the effects of moral context on intentionality ratings.

### Justifications

Consistent with the existence of the naïve concept of intentional action postulated by our multiple-concepts approach, the great majority of justifications for high ratings of intentionality, across contexts and levels of deviance, emphasized the fact that the agent satisfied his intention. For example, in relation to the immoral context characterized by higher intentionality ratings, participants said:

“He intended to break the vase and succeeded. The alternate circumstances are irrelevant.” (Deviance level 1)

“Sam’s intention was fulfilled.” (Deviance level 2)

“That was his intention from the beginning.” (Deviance level 3)

“Sam intended to do this and the events lead to that result.” (Deviance level 4)

“Sam intended to break the vase, and the vase was broken as a result of his actions.” (Deviance level 5)

Likewise, consistent with the existence of the composite concept of intentional action postulated by our multiple-concepts approach, participants across contexts and levels of deviance emphasized the fact that the causal chain did not follow the agent’s plan. For example, within the amoral context characterized by lower ratings of intentionality, participants said:

“He didn’t mean to break the vase with a ricochet. He meant to hit it directly and break it.” (Deviance level 1)

“Fred’s intention was to break the vase but there was no way that he could have intended for all those random things to happen in order the break the vase.” (Deviance level 2)

“He was aiming straight at it and missed—his intention would not have been to break a vase by crashing a car and so forth.” (Deviance level 3)

“While he had the intention of breaking the vase, the means by which it happened was totally random and accidental.” (Deviance level 4)

“Although he did intentionally mean to break the vase, the vase did not break in the way that he had initially and intentionally meant it to.” (Deviance level 5)

In accord with the premise that perceptions of the agent as not having brought about the outcome would lower attributions of intentional action, participants with lower ratings of intentionality often emphasized the fact that the outcome was not an action attributable to the agent by explicitly observing that the agent did not break the vase. These justifications were predominant at higher levels of causal deviance, particularly at the fifth level where, given the causal intervention of the young boy, the breaking of the vase could be easily attributed to another agent. For example, in justifying their low ratings of *intentionality* at the fifth and most extreme level of causal deviance, participants said:

“Again he caused the events, [but] he did not break the vase.” (Amoral context)

“Fred was not the one who broke the vase.” (Amoral context)

“He didn’t do the breaking the little boy did.” (Immoral context)

“Sam intended to break the vase, but ultimately, he did not break it. The young boy broke the vase.” (Immoral context)

“Sam had intention to break the vase, yes, but he didn’t break it.” (Immoral context)

As with the asymmetry in judgments of intentionality, we observed a parallel asymmetry in judgments of action performance in the first four levels of causal deviance.

Consistent with our explanation, participants produced higher ratings of action performance in the immoral context, and often emphasized the fact that the agent had caused the outcome. For example, they said:

“Because yes he did break the vase, just not with the bullet, he was the one that made the car hit the pole and knock the branch down.” (Deviance level 2)

“Yes because no matter how it was done, it got broken because of his actions.” (Deviance level 2)

“He was the root cause.” (Deviance level 3)

“He caused it to happen by shooting the gun.” (Deviance level 3)

“Sam created a chain of events that ended in breaking of the vase.” (Deviance level 4)

“Sam did break the Vase. He was the one who pulled the trigger that broke the vase.” (Deviance level 4)

## Discussion

We have advocated a multiple-concepts approach to the asymmetry in judgments of intentionality observed in mild cases of causal deviance (Sousa and Holbrook, 2010). Here, we experimentally manipulated the degree of mild-to-extreme causal deviance across immoral and amoral contexts. As we predicted, the asymmetry in judgments of intentional action was reduced by the causal deviance manipulation in proportion to the diminution in attributions of blame/credit and of action performance, both of which contribute to the selection of the naïve concept of intentional action^9^. Further highlighting the intrinsic connection between the intentionality asymmetry and perceptions of the actor as to blame/credit and as the performer of the action, the effect of moral context on intentionality attributions was mediated by both blame/credit ratings and (partially) by action performance ratings. Finally, participants’ justifications for their ratings of intentionality and action performance were largely consistent with our overall approach. In sum, the present results show that the asymmetry in intentionality ratings persists in more dramatic extremes of causal deviance, and accord with our multiple-concepts interpretation of the asymmetry.

As well as being consistent with our multiple-concepts approach, the evidence is also a better fit with our approach than with other prominent models.

Modulations of judgments by moral context are typically explained in two basic ways. Some claim that moral considerations *distort* the application of otherwise non-evaluative concepts, making the related judgments depart from some normative standard. For example, Alicke (1992, 2000, 2008) argues that participants’ judgments of agency in immoral contexts are the result of a blame validation mode of processing characterized by the desire to blame the agent, which supposes that their judgments depart from the way one *ought to* judge. Alternatively, Knobe and colleagues claim that moral considerations are *constitutive* of the conceptual competence related to judgments of agency and other closely related judgments (Knobe and Fraser, 2008; Hitchcock and Knobe, 2009; Pettit and Knobe, 2009; Knobe, 2010; but see Knobe’s previous approaches in Knobe, 2003, 2006; Knobe and Burra, 2006). According to this perspective, there is no distortion involved in participants’ judgments of agency in the immoral context—these judgments simply reflect how our conceptual competence normally works, as it is constituted by moral considerations.

Our approach differs from both of these perspectives in several respects. First, our multiple-concepts approach provides an alternative explanation of the asymmetry in judgments of intentionality. In contrast with Alicke’s explanation, the multiple-concepts account does not entail distortion in the judgments of intentionality of our participants, let alone a distortion driven by moral considerations. Rather, participants tend to bring to bear the naïve concept in immoral contexts because it is most salient given the importance of the immoral intentions to considerations of moral blameworthiness. This probabilistic bias in concept selection should not be conflated with the purported distortion of the application of a single concept of intentional action (Note that our point here stands even if our approach is interpreted in terms of hybridism—see text footnote 2). Given that there is no convincing normative standard from which participants’ answers in the immoral context depart (see also Sousa and Holbrook, 2010), we believe the multiple-concepts approach provides a better explanation than Alicke’s. Moreover, in our current results the effect of moral context on intentionality attributions was partially mediated by action performance ratings, suggesting that participants are deploying their conceptual competence in a logically coherent way, not in a way that departs from some normative standard.

Now, while our explanation of the asymmetry gives prominence to blame considerations, Knobe’s current account of the constitutive influence of moral considerations excludes any reference to blame: “… the account makes no mention at all of blame” (Knobe, 2010, p. 328). Thus, while our account predicts our current results showing that the effect of moral context on intentionality attributions was fully mediated by blame/credit ratings, Knobe’s account is not consistent with these results. Also, although Knobe has specified how his account could explain the intentionality asymmetry in the context of side-effects, it is doubtful that his account can explain the intentionality asymmetry in the types of lucky contexts related to our results (see Cova, 2015). Finally, Knobe has criticized those who postulate a polysemy to explain the asymmetry in judgments of intentionality by saying that this type of approach could not lead to a unified explanation of the range of moral asymmetries found in the current literature, as one would have to postulate an *ad hoc* polysemy for each of the asymmetries, which seems quite implausible. However, we do not see any good reason for pursuing a unified explanation for all the moral asymmetries found in the literature (Hindriks, 2014; see also Sousa and Mauro, 2015).

The second important difference between our approach and the accounts offered by Alike and Knobe relates to our explanation of the asymmetry in attributions of action performance. Both of the alternative perspectives suggest that, akin to their accounts of the asymmetry in judgments of intentionality, blame motivations or constitutive moral considerations would explain the asymmetry in attributions of action performance (see also related discussion in Reuter et al., 2014). Our explanation for the inflated ratings of action performance in the immoral context as owing to increased causal salience due to blame considerations does not seem consistent with either Alicke’s or Knobe’s perspective, as our explanation entails simply that the general concept of action is highly underspecified and hence susceptible to a variety of contextual specifications driven by different factors (Note that we are not postulating polysemy in relation to the folk concept of action). Contrary to Alicke’s approach, there are no evident normative standards that judgments of action performance would be violating in the immoral context. Contrary to Knobe’s approach, moral considerations do not seem plausibly built into the general concept of action. However, further research on the structure of the general concept of action, and of the determinants of whether event sequences are categorized as coherent actions, is required to understand the asymmetry documented here, and the plausibility or compatibility of these different explanations.

Falkenstien (2013) replicated the asymmetry in intentionality judgments dealing with cases of luck due to lack of skill and provided a different type of explanation that, as her model suggests, could be potentially extended to both of our agency asymmetries. Falkenstien utilized a version of Knobe’s (2003) original skill scenarios, which are quite similar to the scenarios we described initially, except that they do not include the ricochet aspect:

Jake desperately wants to win the rifle contest [to have more money]. He knows that he will only win the contest if he hits the bulls-eye. [He knows that he will inherit a lot of money when his aunt dies. One day, he sees his aunt walking by the window.] He raises the rifle, gets the bull’s-eye [her] in the sights, and presses the trigger. But Jake isn’t very good at using his rifle. His hand slips on the barrel of the gun, and the shot goes wild… Nonetheless, the bullet lands directly on the bull’s-eye [hits her directly in the heart]. Jake wins the contest [She dies instantly].

The explanation proposed by Falkenstien to the asymmetry in intentionality judgments related to the above scenarios is based on the idea that these scenarios lead participants to raise different types of questions, and that these questions influence their ratings of intentionality. According to her, the scenarios influence the questions that participants consider in the following way:

When the sharp-shooter shoots at a target [the bull’s-eye], it seems irrelevant to ask, “Why did he want to hit it?” After all, wouldn’t anyone in his position have done the same thing? It seems much more interesting to ask, “How did he manage to succeed?” since it is rather surprising that he won, given his lack of skill. But when the sharp-shooter shoots at his aunt, a question like “Why did he want to shoot her?” suddenly seems very relevant; in addition to wondering how he succeeded, a reader also probably wonders what made him do such an awful thing. (Falkenstien, 2013, p. 298)

According to Falkenstien, these divergent questions have the following downstream effects on participants’ perceptions of intentionality:

(…) when observers focus on questions that draw attention to the actor’s mental states, they are more likely to be aware of the actor’s intentions and thus find the action intentional. For example, when the relevant question is, “How did the actor manage to succeed?,” the answer doesn’t invoke the actor’s intentions at all. He succeeded because he was lucky. The circumstances, not his intentions, answer the question. However, when the relevant question is “Why did he shoot at his aunt?,” it draws attention to the actor’s choice to act the way he did. That kind of question forces the observer to notice the importance of the actor’s decision (above and beyond his circumstances): the event hinged on the decision of the actor. That subconscious consideration of the actor’s intent, drawn out through consideration of certain questions, makes people judge the action to be intentional. (Falkenstien, 2013, p. 298)

This explanation does not seem plausible. First, Falkenstien does not provide any evidence that participants consider the why-question in the context of the immoral scenario and the how-question in the context of the amoral scenario, and, at least in relation to the immoral scenario, it appears doubtful that the why-question would be raised in the minds of participants, since the scenario explicitly states about the motivation of the agent. Given that the immoral scenario is fairly explicit about the intention to kill the aunt for inheritance money, why would a participant raise a why-question concerning the motive of the shooting?

Moreover, Falkenstien does not provide any evidence for her claim that participants who rate the action as unintentional do not take into account the mental states of the protagonist, focusing only on a type of luck that is independent of considerations of mental states and/or on the lack of skill qua a dispositional property of the agent. Actually, in our previous research, we also probed scenarios similar to Knobe’s original scenarios (i.e., scenarios without a ricochet), and most participants justified their answer that the action was unintentional in terms of causal deviance, which includes considerations of mental states (Sousa and Holbrook, 2010; see also text footnote 1). For example, they said:

Jake may have been intending to hit the bull’s-eye, but instead he slipped and got lucky. The slip was unintentional, and therefore the shot resulting from it was also unintentionally aimed.

…because his hand slipped and he didn’t mean to fire the gun at that point in time.

In other words, most participants interpreted these skill scenarios in terms of a departure from the *plan* of the agent—the goal was satisfied but not *in the way intended*.

Finally, our multiple-concepts approach furnishes an arguably better explanation of Falkenstien’s own results, which undermines the plausibility of extending her model to explain our results. In her first two studies, she found that when participants were explicitly asked which question, the why- or how-question, seemed more relevant to the immoral versus amoral scenarios, they tended to pick the why-question in the immoral scenario and the how-question in the amoral one. However, this can be easily explained in terms of participants’ concerns with immoral blame and amoral praise, as we have discussed. More importantly, in her last study, she manipulated the questions by priming participants with the why- or how-question in relation to the immoral and amoral scenarios (i.e., by making participants think about either the why- or how-question in relation to each of the scenarios), finding that this manipulation *only* influenced ratings of intentionality in the amoral scenario. While this result conflicts with her explanation, it can be readily explained by our model. Only the naïve concept is relevant for most participants in the immoral context—hence, the null effect of question priming—whereas both concepts are salient to participants in the amoral context—hence, the effect of question priming (For more detailed evidence on the fact that both concepts are salient in the amoral context, see Sousa and Holbrook, 2010). In sum, our approach appears to accommodate both the present data and Falkenstien’s own results.

### Limitations and Future Directions

Our data provide clear grounds to conclude that the asymmetry in judgments of intentionality is robust to manipulations of all but the most extreme degrees of causal deviance, and that the asymmetry is contingent on attributions of blame/credit and action performance. These findings can be explained by our multiple-concepts approach. However, the results must be considered preliminary pending further research that addresses limitations of the present work.

Building on the present “proof-of-concept,” future studies should examine more precisely the different variables that may influence perceptions of action performance. For example, investigators should manipulate both the number and temporal duration of the causal steps intervening between the initial action of the agent and the final effect of the causal chain. Plausibly, the most determinative variable will be that of the involvement of other agents. Our findings suggest that the voluntary interference of another human agent (as in our most extreme causal deviance condition) constitutes a clear boundary condition with regards to the perception of the outcome as stemming from the initial agent. Follow-up studies might interpose the involvement of an agent earlier in the sequence of events—both asymmetries may well have been eliminated had we depicted the boy as having broken the vase much earlier in the unfolding causal chain. Indeed, the voluntary interference of another human agent may cancel the understanding of the event as an action of the original agent quite independent of other variables, for, at least under certain circumstances, people may accord a very special causal role to voluntary actions (for related discussions, see Hart and Honore, 1959; Feinberg, 1970b; Hilton et al., 2009).

We have highlighted the role of moral context, empirically contrasting immoral and amoral scenarios. Future extensions of this research should assess asymmetries with regard to actions that are morally praiseworthy, such as a condition in which the agent’s motivation to win the contest was to use the prize money to save an orphanage. Our multiple-concepts account produces the same predictions with regard to immoral and to positive moral contexts: to the extent that moral intentions are more relevant in the appraisal of morally praiseworthy acts than are the means by which they are carried out, such contexts should make salient the naïve concept of intentionality, driving an asymmetry in judgments of intentionality (cf. Knobe, 2003).

Finally, our participant sample was drawn from the United States, one of the most non-representative societies in the world concerning many fundamental psychological dimensions (Henrich et al., 2010). Moreover, it is plausible to suppose that competencies involving causal cognition are susceptible to cultural elaborations, and that there is a significant degree of cultural diversity in their deployment (see, e.g., Morris and Peng, 1994; Bender and Beller, 2011). Replication with other samples and languages is therefore required before drawing more general conclusions concerning the issues at stake here.

Although we presume that in every human society people will have concepts of action, acting intentionally, and blame/credit, there may indeed be different cultural elaborations of these concepts and their interrelations. Anthropologists have claimed that, in many cultural contexts, people adhere to an opacity-of-other-minds folk doctrine that proscribes the ascription of intentions either in itself or as a factor in blame attribution (Rumsey and Robbins, 2008; see also Wassmann et al., 2013). We are skeptical about strong relativist interpretations of this opacity doctrine. For example, we are skeptical about the idea that in some of these contexts people do not read other minds (see Astuti, 2012), or in no way take into account intentionality or its absence when attributing culpability and liability, adhering therefore to a doctrine of strict or absolute liability (see Goldman, 1993; Astuti and Bloch, 2015; Sousa and Manoharan, forthcoming). However, the existence of cultural norms downplaying ascriptions of intentionality may indeed lead to important cultural differences in relation to our topic. Thus, one interesting direction of investigation would be to probe whether people use distinct concepts of intentional action in these “opacity” contexts in connection with different sorts of blame/credit attributions, and whether the asymmetries we found in our results would be replicated.

There is also the related issue of variation concerning the relationship between language and concepts. Although we accept that many concepts are not linguistically encoded (see Sperber and Wilson, 1998), we expect that, given their relevance to human interaction, concepts of action, acting intentionally, and blame/credit will be encoded, by lexical or other grammatical means, in most, if not all, languages. Thus, another interesting line of research would be to probe whether one would find linguistic structures in other languages that evince a polysemy similar to that of “acting intentionally” in English, although, as one of the reviewers correctly pointed out, there are real translation challenges when one moves to more distant languages, like polysynthetic languages.

## Conclusion

In this article, we focused on causal cognition as deployed in judgments of agency, dealing with a familiar Western context. Our investigation indicates a complex picture. The language of action is vague in that it expresses a concept that is underspecified, while the language of intentional action is polysemous in that it expresses different concepts (for additional concepts not discussed in this article, see Nichols and Ulatowski, 2007; Sousa and Holbrook, 2010, footnote 10; Cova, 2015). The relevance of different concepts is connected to moral considerations.

Many anthropologists have argued that a necessary condition for a good understanding of the extent of cultural variation in relation to any aspect of human cognition is a fine-grained understanding of the aspect in one’s own culture. We take this article as a contribution in this direction, and we hope that our findings are taken into account in pursuing cross-cultural research on the topic of causal cognition and agency.

### Conflict of Interest Statement

The authors declare that the research was conducted in the absence of any commercial or financial relationships that could be construed as a potential conflict of interest.

## Footnotes

1 This type of case was discussed initially in terms of the relevance of a skill component to the folk concept of intentional action (see Knobe, 2003; Malle, 2006; see also Malle and Knobe, 1997). However, we have shown that, in these types of scenarios, what people see as relevant for judging that the action of hitting the target is unintentional is the presence of causal deviance rather than the absence of skill qua a dispositional property of the agent (see Sousa and Holbrook, 2010).

2 We have construed the polysemy of “intentional action” in terms of the expression of different concepts—a pluralist perspective. However, one could easily reinterpret our discussion of the polysemy at stake here in terms of the expression of different senses that are aspects of the *same* concept—a hybridist perspective. To decide between these two perspectives in relation to our topic, one would need a consensual criterion to individuate concepts, an issue without a clear resolution in the psychology of concepts (see Machery, 2009; Vicente and Manrique, 2014; see also Pustejovsky, 1995; Wilson and Carston, 2007). Thus, although we are phrasing our approach in pluralistic terms, we are open to a hybridist interpretation.

3 Our characterization here is inspired by work on thematic relations and semantic macroroles in Semantics (in particular, see Jackendoff, 1990, chapter 7; Jackendoff, 2007, chapters 6 and 8; see also Van Valin and Wilkins, 1996). For discussions of cross-linguistic differences concerning constraints on the type of entity that may be understood as a causal producer, see Wolff et al. (2009) and Kanero et al. (2015).

4 For some cross-linguistic differences in this regard, see Fausey et al. (2010) and Fausey and Boroditsky (2011).

5 For related discussions, see Feinberg’s (1970a) remarks about the accordion effect of action descriptions, work on lexical and periphrastic causatives (e.g., Fodor, 1970; Wolff and Gentner, 1997; Dixon, 2000; Wolff et al., 2009), and work on event segmentation (e.g., Bohnemeyer et al., 2010).

6 It is important to note that while Mary’s death is a causal consequence of the action *opening the door*, it is an effect internal to the action *killing Mary*. In other words, while one could say, “John caused Mary’s death by opening the door,” one could not say, “John caused Mary’s death by killing Mary” (cf. Goldman, 1970).

7 A similar issue arises in the legal system when, in cases where the (intended) causation of death involves extreme causal deviance, one tries to argue that the crime is one of attempted murder instead of murder (for a related discussion, see Sousa, 2009).

8 The distributions for all three ratings significantly differed between the amoral and immoral context condition due to the right-skewed pattern of judgments produced in the immoral condition. Accordingly, non-parametric Mann–Whitney U tests were conducted as well, confirming that the effects of moral condition were highly significant for all three ratings, *p*s < 0.001.

9 Although blame ratings shifted downward with increasing degrees of causal deviance, they remained quite high even at the most extreme level. This is consistent with our hypothesis that attributions of moral blame focus primarily on immoral intentions (see also Sousa, 2009).
